# Dual roles for the ER membrane protein complex in flavivirus infection: viral entry and protein biogenesis

**DOI:** 10.1038/s41598-019-45910-9

**Published:** 2019-07-04

**Authors:** Nicholas J. Barrows, Yesseinia Anglero-Rodriguez, Byungil Kim, Sharon F. Jamison, Caroline Le Sommer, Charles E. McGee, James L. Pearson, George Dimopoulos, Manuel Ascano, Shelton S. Bradrick, Mariano A. Garcia-Blanco

**Affiliations:** 1 Department of Microbiology and Molecular Genetics, and Center for RNA Biology, Duke University, Durham, USA; 2 Department of Biochemistry and Molecular Biology, University of Texas Medical Branch, Galveston, USA; 3 W. Harry Feinstone Department of Molecular Microbiology and Immunology, Johns Hopkins Bloomberg School of Public Health, Baltimore, USA; 4 Department of Biochemistry, Vanderbilt University, Nashville, USA; 5 Duke Human Vaccine Institute, Duke University, Durham, USA; 6 Programme of Emerging Infectious Diseases, Duke-NUS Medical School, Singapore, Singapore

**Keywords:** Dengue virus, Endoplasmic reticulum

## Abstract

Hundreds of cellular host factors are required to support dengue virus infection, but their identity and roles are incompletely characterized. Here, we identify human host dependency factors required for efficient dengue virus-2 (DENV2) infection of human cells. We focused on two, TTC35 and TMEM111, which we previously demonstrated to be required for yellow fever virus (YFV) infection and others subsequently showed were also required by other flaviviruses. These proteins are components of the human endoplasmic reticulum membrane protein complex (EMC), which has roles in ER-associated protein biogenesis and lipid metabolism. We report that DENV, YFV and Zika virus (ZIKV) infections were strikingly inhibited, while West Nile virus infection was unchanged, in cells that lack EMC subunit 4. Furthermore, targeted depletion of EMC subunits in live mosquitoes significantly reduced DENV2 propagation *in vivo*. Using a novel uncoating assay, which measures interactions between host RNA-binding proteins and incoming viral RNA, we show that EMC is required at or prior to virus uncoating. Importantly, we uncovered a second and important role for the EMC. The complex is required for viral protein accumulation in a cell line harboring a ZIKV replicon, indicating that EMC participates in the complex process of viral protein biogenesis.

## Introduction

The Flavivirus genus includes three arboviruses transmitted by *Aedes* species of mosquitoes that are important human pathogens: dengue (DENV 1–4), yellow fever (YFV) and Zika (ZIKV) viruses. DENV1-4 cause 100 million cases of dengue fever and, less commonly, severe diseases including dengue hemorrhagic or dengue shock syndromes, each year^1^. The burden of yellow fever on global health is hard to estimate, however, recent outbreaks in Africa and Brazil highlight the continued importance of this virus that causes severe disease^2^. The recent outbreak of ZIKV in the Americas revealed an association with Guillain-Barre syndrome among adults and the danger of fetal infection^3–7^. Universal countermeasures to combat the spread of these viruses are limited to preventing contact between mosquitoes and humans using public health approaches. Preventive vaccination is only truly effective for yellow fever, and only somewhat effective for dengue viruses^8–10^. No anti-viral treatments are approved to treat infected individuals nor prevent infection of at-risk populations. Therefore, there exists significant need for a better understanding of the biology of these *Aedes* transmitted flaviviruses in the hopes this understanding will provide new ways to counteract them.

Flaviviruses enter the cell by recognizing one or more receptors at the cell surface followed by receptor mediated endocytosis^9^. Virus is trafficked to a late endocytic compartment where acidification by the cellular vacuolar ATPase leads to structural changes in the viral envelope protein, fusion of the endosomal and viral membranes, and release of the viral genome into the cell cytoplasm. The capped, non-polyadenylated positive sense RNA viral genome is translated from a single open reading frame into a viral polyprotein. The synthesis of the viral polyprotein and its co- and post-translational processing takes place in close association with the endoplasmic reticulum (ER) and mature viral proteins are found in the ER lumen and ER membranes and also in the cytosol^11^. Flaviviruses, like all viruses, use many host translation factors to synthesize their proteins^12^ and equally subsume the ER-associated cellular machinery to localize proteins in membranes and to secrete them^13^. Unbiased, systematic efforts have identified hundreds of candidate genes required for viral infection (proviral factors) including many involved in translation and co-translational processing^9,14–21^.

Among the proviral factors identified by these screens were subunits of the ER membrane protein complex (EMC). Our lab reported the first glimpse that the EMC was an important flavivirus proviral factor by observing that small interfering RNAs (siRNA) targeting EMC2 (identified as TTC35) and EMC3 (identified as TMEM111) reduced YFV17D infection^16^. Wu and colleagues reported that WNV replication kinetics were delayed in an EMC2 knockout cell line, although ultimately the EMC was dispensable for WNV virus production^17^. Subsequently, three more teams identified the EMC as an important proviral factor for DENV, YFV and ZIKV infection^18–20^. The study by the Brass lab suggested that EMC knockdown did not affect viral attachment, but implied that viral entry and/or replication may have been impeded through an unidentified mechanism^19^.

The EMC is a highly conserved, ER-localized, heterodecameric protein complex composed of subunits EMC1-7, 8a, 8b and 10^22,23^. Schuldiner and colleagues first described the EMC and speculated that the complex may promote folding of membrane proteins^24^. Recently, the EMC was shown to direct the insertion of transmembrane domains consistent with a role in protein biogenesis^25,26^. These studies suggest that the diverse phenotypes observed after experimental modulation of the EMC are due to altered accumulation, maturation and/or folding of proteins that transit through or are associated with the ER.

Here, we describe a genome-scale RNAi loss of function screen in human cells that confirms the role of the EMC in DENV2 replication. We show that the EMC is required for effective DENV2 infection of *Aedes aegypti* mosquitoes, further emphasizing the importance of this complex. The critical requirement of the EMC for DENV2, DENV4, YFV, and ZIKV replication was validated in EMC4 knock out cells. In agreement with published reports, we observed an early block to DENV2 replication characterized by reduced accumulation of viral protein in cells lacking EMC4. Indeed, using a novel assay to measure interactions of incoming viral genomes with cellular proteins, we observed that EMC is required for virus uncoating or a step prior to it. Furthermore, using cells persistently harboring a ZIKV replicon, we show that the EMC is also necessary for a later step in the viral lifecycle since biogenesis of the viral non-structural proteins was impaired by knockdown of EMC subunits. This proposed mode of action on viral protein biogenesis is consistent with known effects of the EMC on ER associated cellular proteins^24,25,27–29^. Thus, the EMC is a critical host factor that acts at multiple stages of the flavivirus lifecycle.

## Results

### A genome-scale RNAi screen identifies DENV host factors

**Figure 1:**
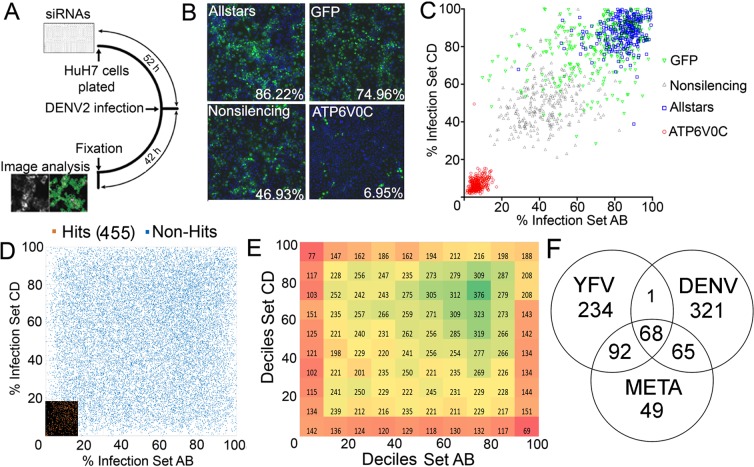
An RNAi based screen for DENV2 human host factors. (**A**) Schematic describing the RNAi screen work flow. (**B**) Representative images from control wells. Infected cells are green and nuclei are blue. Rates of infection across the screen are indicated for each control. (**C**) Behavior of all control wells across the screen in terms of infection percentage. (**D**) Each point represents infection rate for wells AB and CD for a given gene. The 455 hits are highlighted in lower left corner. (**E**) Same data as in panel D plotted in deciles. The color represents numbers of genes in each square. (**F**) Venn diagram indicating numbers of host factor genes commonly identified between DENV and YFV RNAi screens, and identified by meta-analysis of both screens (see Materials and Methods).

We conducted a genome-scale RNAi screen to identify host proteins that are necessary for robust DENV2 (New Guinea C strain) infection using a siRNA library targeting 22,909 mRNAs in human HuH-7 hepatoma cells (Fig. 1A). HuH-7 cells were reverse-transfected with siRNAs, incubated for 52 hours and inoculated with DENV2 at low multiplicity of infection (MOI). At 42 hours post-infection, cells were fixed and stained using an anti-flavivirus envelope protein antibody and Hoechst stain^30^. The fraction of cells infected in each well was ascertained by high-content imaging and analysis (Fig. 1A). Each plate had three independent negative control siRNAs (AllStars, Nonsilencing and GFP) and a positive control siRNA targeting a subunit of the vacuolar ATPase (ATP6V0C), which is required for endosomal acidification and efficient virus infection^15,16,30,31^. Figure 1B shows individual fields and percent infection for the average from the population of the negative (AllStars = 86.22% infected, Nonsilencing = 46.93% infected, and GFP = 74.96% infected) and positive control (ATP6V0C = 6.95% infected) siRNAs. The distribution of the four control siRNAs throughout the screen is shown in Fig. 1C and in Table S1. The significant differences between the three negative control siRNAs likely reflect different off-target effects for each of the siRNAs. Importantly, however, all three negative controls were significantly different and easily distinguished from the ATP6V0C positive control (One-way ANOVA, P < 0.0001, Tukey’s post-test, P < 0.01).

In the screen, each gene product was targeted by at least four unique siRNAs grouped into two distinct pools (Set AB or Set CD), generating two separate measurements of infection rate for each gene (Fig. 1D)^15,16,30^. The complete experimental data for the screen are presented in Table S1. The distribution of infection rates spanned the range of the assay (0 to 100%) with the majority of data points skewed towards higher rates of infection (Fig. 1E), suggesting that our screen could effectively identify hits as strong as the positive control. We identified 455 hits that had a Z-score less than or equal to 3 relative to the ATP6V0C siRNA positive controls (see “hits” tab in Table S1). Similar to a reported RNAi screen for YFV17D host factors^16^, the most potent candidate host factors identified in this screen included ribosomal proteins (predominantly of the 60S subunit), components of the vacuolar proton pump (e.g., ATP6V0D), proteins required for protein translocation across the ER (e.g., SEC. 61A1), and proteins known to participate in biogenesis of flavivirus proteins (e.g., SPCS2). Among very interesting novel hits is the poorly annotated RNA-binding domain containing protein C1ORF144, which associates with SRP9 and SRP14^32^, two components of the signal recognition complex. Importantly, EMC3, EMC2 and EMC5 were identified as potential DENV2 proviral host factors (Table S1). Identification of these known and suspected flavivirus host factors suggested that the DENV RNAi screen was robust.

### Meta-analysis of DENV and YF17D RNAi screen identifies novel candidate Flavivirus proviral factors

The screen for DENV proviral factors reported herein was performed with the same siRNA library, cell line and primary antibody as a previously published screen for YFV17D proviral factors^16,33^, permitting a robust meta-analysis of the two screens. The YFV and DENV screen datasets were aligned, and after 2,034 genes were removed due to low cell number in any of the interrogated wells, we analyzed the data for 20,875 genes. The percent of cells infected by either YFV17D or DENV2 was ranked, and a nonparametric summation of ranks was adopted that permitted comparison between the screens. A permutation analysis of the ranks was performed and identified a p ≤ 0.00137 to give a false discovery rate of less than 30 genes. Using this criterion, we identified 274 common proviral factors (Table S2), 49 of which had not been identified by either screen alone (Fig. 1F).

The meta-analysis reinforces some concepts that have emerged from previous screens. First, there is a dramatic overrepresentation of proteins of the large ribosomal subunit: 21 canonical large subunit proteins vs 6 proteins of the small ribosomal subunit (see large ribosomal subunit proteins in red font and small subunit proteins in purple font in Table S2). Additionally, we found several subunits of the vATPase and factors involved in translation of ER resident proteins (SEC61, SRP54).

Among the 274 common hits were TTC35, TMEM111 and TMEM32 (MMGT1/EMC5) which are EMC2, EMC3 and EMC5, respectively^23^. No other EMC subunit was identified by our siRNA screens. Interestingly, the YFV17D proviral factor hit list identified EMC2 and EMC3 but did not pick up EMC5, however, the meta-analysis, combining the power of testing related viruses using a common screening paradigm, suggested that EMC5 is also a proviral factor for YFV17D. The meta-analysis also predicted that C1orf9, which is the human homologue to yeast Slp1p, a protein that was suggested to have a role in the yeast EMC pathway^24^ is a host factor for DENV2 and YFV. Consistent with our YFV screen^16^ and more recent screens of others^18–20^, our data identified a subset of the EMC subunits and a protein associated with the EMC pathway as YFV and DENV2 candidate host factors. Given that the EMC was observed to strongly impact several flaviviruses we decided to study this complex further.

### Validation of EMC as a proviral factor

**Figure 2:**
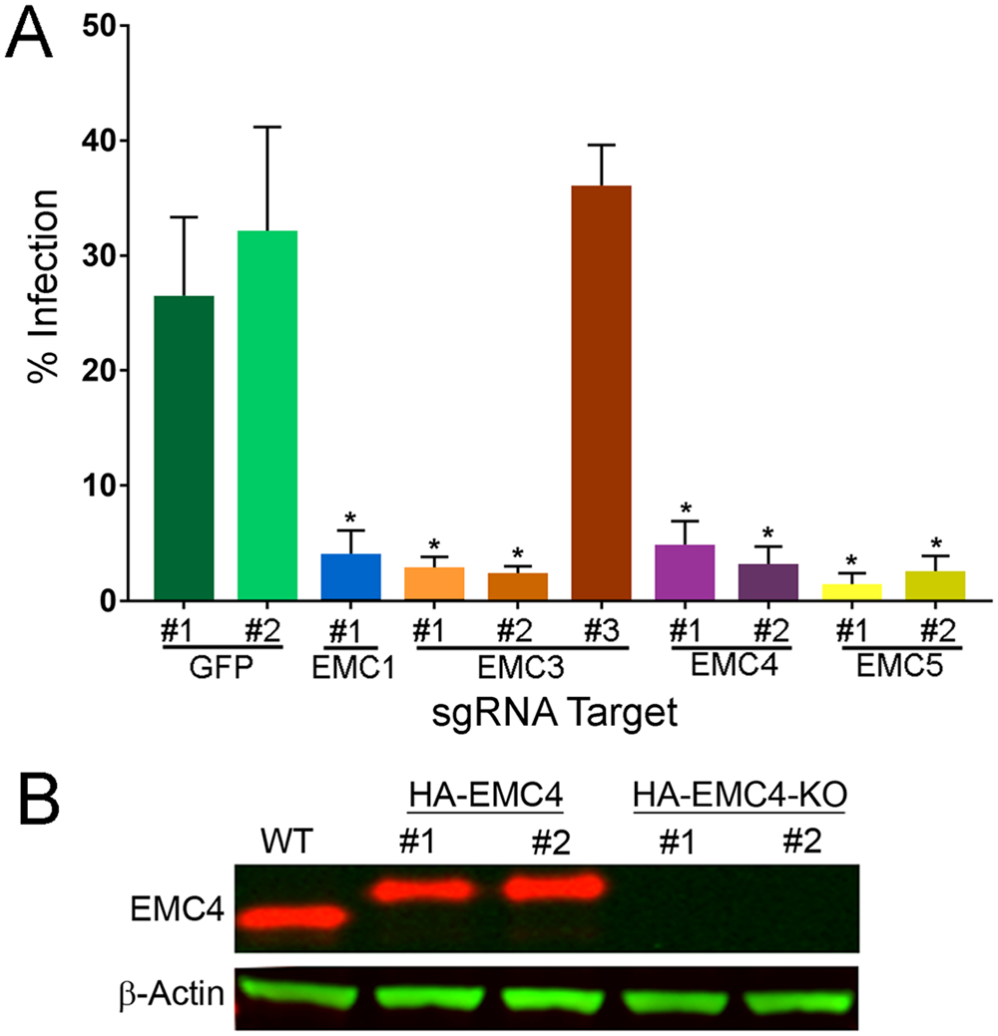
Validation of EMC as a proviral factor for DENV2 and establishment of EMC4-modified cell lines. (**A**) Cells were transfected with sgRNAs to knockout the indicated genes in HuH-7 cells. Subsequently cells were infected with DENV2 and rates of infection measured by high content imaging. sgRNAs targeting GFP served as negative controls. (**B**) Western blot characterization of clonal, gene-edited HuH-7 lines. Cells containing an HA-tag knocked into EMC4 alleles were initially generated and a single clone was used to generate two clonal HA-EMC4 knockout lines. Levels of other EMC subunits are shown for each cell line. WT indicates parental HuH-7 cells. (*p < 0.05). The western blots are from the same gel and the image was cropped to show EMC4 and HA-EMC4, and ß-actin (loading control).

We initially validated the YFV screen results using gene editing technology^34^ by transfecting pools of HuH-7 cells with plasmids expressing Cas9 and sgRNAs targeting various EMC subunit genes and infecting with YFV17D at a MOI 0.1. Approximately 42 hours post infection (pi), a time point that allows for multiple viral lifecycles, the infection was terminated and cells were labeled by immunofluorescence targeting the viral E protein. The fraction of cells positive for E protein (% infected) was quantified by automated imaging. Relative to the two GFP controls, one sgRNA targeting *EMC1*, and two independent sgRNAs each targeting *EMC3, EMC4*, or *EMC5* reduced infectivity 5- to 20-fold (Fig. 2). While we do not know why EMC3 sgRNA#3 failed to inhibit, we conjecture that this sgRNA failed to edit the EMC3 locus. The combined results from the RNAi-based screens and gene editing validation experiments established EMC1-5 as YFV proviral host factors. The requirement for many subunits of the EMC for efficient infection strongly suggested that the entire human EMC is a flavivirus proviral factor, and is consistent with the model, first proposed by Schuldiner and colleagues, that the EMC is a multi-protein complex and loss of an individual subunit disables the entire complex^24^.

We used gene editing to establish HuH-7 lines where EMC4 was tagged with an N-terminal HA epitope (Figs 2B and S1 for uncropped gel). Two independent *EMC4* null cell lines were then generated from one of the HuH-7(HA-EMC4) clones and selected for further analysis. Endogenous EMC2 was expressed at equivalent levels in all cell lines while EMC4 was modified with the HA tag or knocked out. Importantly, the parental HuH-7(HA-EMC4) and daughter HuH-7(HA-EMC4 KO) cell lines were propagated similarly over many passages and were morphologically indistinguishable, demonstrating that the EMC is not essential for HuH-7 cell viability and proliferation.

**Figure 3:**
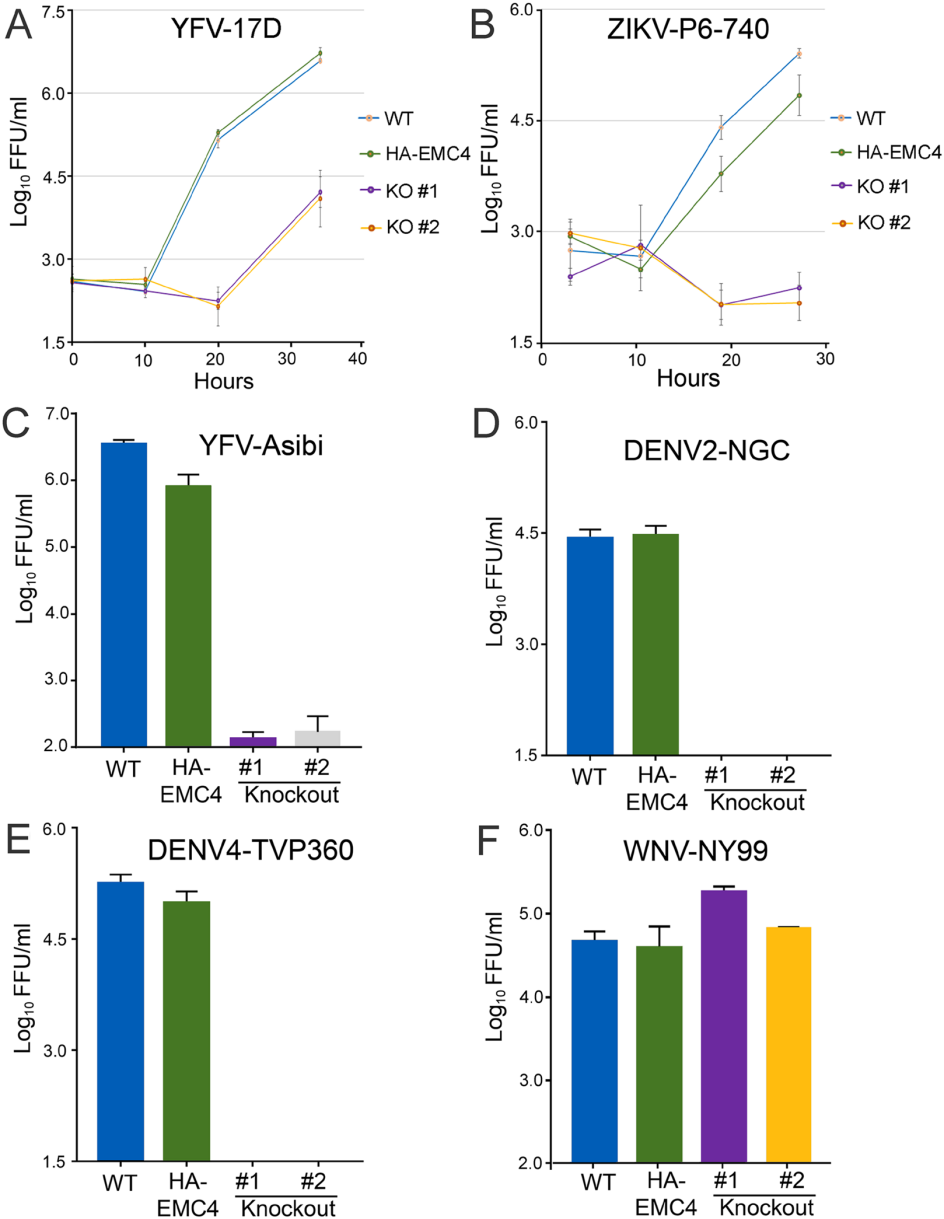
EMC4 knock out causes striking inhibition of DENV2, DENV4, YFV, ZIKV replication. (**A**) YFV17D virus produced by genetically modified cell lines infected at an MOI 1 is shown. Virus-containing media was harvested at 0, 9.8, 20 and 33.5 hours pi. Data shown are representative of 3 independent assays. The limit of detection is 1.5 log_10_(FFU/mL). (**B**) ZIKV virus produced by genetically modified cell lines infected at an MOI 10 is shown. Virus-containing media was harvested at 3, 10.5, 19 and 27.3 hours pi. This assay was performed one time. The limit of detection for this assay is 1.5 log_10_(FFU/mL). (**C**) YFV Asibi virus produced by genetically modified cell lines infected at an MOI 1 is shown. Virus-containing media was harvested 42 hours pi. Data are representative of 2 independent assays. The limit of detection for this assay is 1.7 log_10_(FFU/mL). (**D**) DENV2 virus produced by genetically modified cell lines infected at an MOI 5 is shown. Virus-containing media was harvested at 42 hours pi. Data shown are representative of 3 independent assays. The limit of detection for this assay is 1.5 log_10_(FFU/mL). (**E**) DENV4 virus produced by genetically modified cell lines infected at an MOI 2 is shown. Virus-containing media was harvested at 42 hours pi. Data shown are representative of 2 independent assays. The limit of detection for this assay is 1.5 log_10_(FFU/mL). (**F**) WNV virus produced by genetically modified cell lines infected at an MOI 0.5 is shown. Virus-containing media was harvested 48 hours pi. This assay was performed one time. The limit of detection for this assay is 1.7 log_10_(FFU/mL). Each bar or time point represents the mean and standard deviation for 3 replicate wells. Abbreviations: WT = HuH-7(WT), HA-EMC4 = HuH-7(HA-EMC4) cell line, KO #1 and KO #2 = HuH-7(HA-EMC4 KO) cell lines clones 1 and 2.

We next quantified production of extracellular virus in HuH-7(WT), HuH-7(HA-EMC4) and HuH-7(HA-EMC4 KO) cell lines infected with multiple flaviviruses. We analyzed the kinetics of infectious virus production for YFV17D and ZIKV (P6-740) viruses. For YFV, the initial viral burst occurred by 20 hr post-infection (pi) in the HuH-7(WT) and HuH-7(HA-EMC4) cell lines and virus production continued to increase up to 33.5 hr pi (Fig. 3A). In the EMC4 KO cell lines virus production was significantly delayed with no increase at 20 hr pi and up to 3 log_10_ reduction in virus titer relative to the parental HuH-7(WT) and HuH-7(HA-EMC4) cells lines at 33.5 hr pi (Fig. 3A). For the Asian lineage ZIKV (P6-740), we detected 5.4 log_10_ FFU/mL or 4.8 log_10_ FFU/mL in HuH-7(WT) and HuH-7(HA-EMC4) cell lines, respectively; however, no increase in infectious ZIKV was observed over the time course in EMC4 KO cells (Fig. 3B), indicating that EMC4 was absolutely required for productive ZIKV infection.

Additionally, we screened a panel of flavivirus strains at single time points after infection. We asked if EMC was required for efficient replication of the pathogenic YFV Asibi strain, which was used to derive the 17D strain^35^, and observed that HuH-7 and HA-EMC4 cells produced over 3 log_10_ FFU/mL more YFV Asibi than EMC4 KO cell lines (Fig. 3C). These data established the EMC as a critically important proviral factor supporting YFV replication in human cells. We analyzed infection of DENV strains from two different serotypes in EMC4 KO cell lines. DENV2-NGC and DENV4-TVP360 replicated to approximately 4 or 5 log_10_ FFU/mL, respectively, in HuH-7(WT) and HA-EMC4 cell lines, but virus production was reduced to below detectable levels in the two EMC4 KO cells lines (Fig. 3D,E), validating EMC as proviral factor for DENV. Finally, we tested the distantly related virus WNV, which, unlike DENV, YFV and ZIKV, is transmitted by *Culex* rather than *Aedes* mosquitoes. Work of Ma and colleagues^17^ suggested that WNV did not required EMC for viral replication. Indeed, pathogenic WNV-NY99 virus production by EMC4 KO cells lines was similar to that for the control parental cell lines (Fig. 3F). Together, these results indicated that the EMC is required by pathogenic flaviviruses transmitted by *Aedes* mosquito species. The magnitude of the effects observed for DENV2, DENV4 and ZIKV was impressive and indicated an essential role for the EMC in the replication of these viruses.

### The EMC is required for efficient infection of *Aedes aegypti* mosquitoes

Previously, we reported a large overlap between dipteran and human proviral factors^15^ and we wondered whether the EMC is a proviral factor in mosquitoes. The EMC is highly conserved between human and *Aedes aegypti*, which is the principal insect vector for urban cycles of YFV, ZIKV and DENV^4,36^. *Aedes aegypti* EMC subunits EMC2, EMC3 and EMC4 were readily identified. EMC2 (aaEMC2; XP_001661937), EMC3 (aaEMC3; XP_001652133), and EMC4 (aaEMC4; XP_001657467) are 44%, 65% and 51% identical based on protein sequence comparison to the respective human EMC subunits EMC2 (NP_055488), EMC3 (NP_060917), and EMC4 (NP_057538). These three EMC subunits were selected for RNAi mediated depletion using an established model of knockdown in mosquitoes^37^.

**Figure 4:**
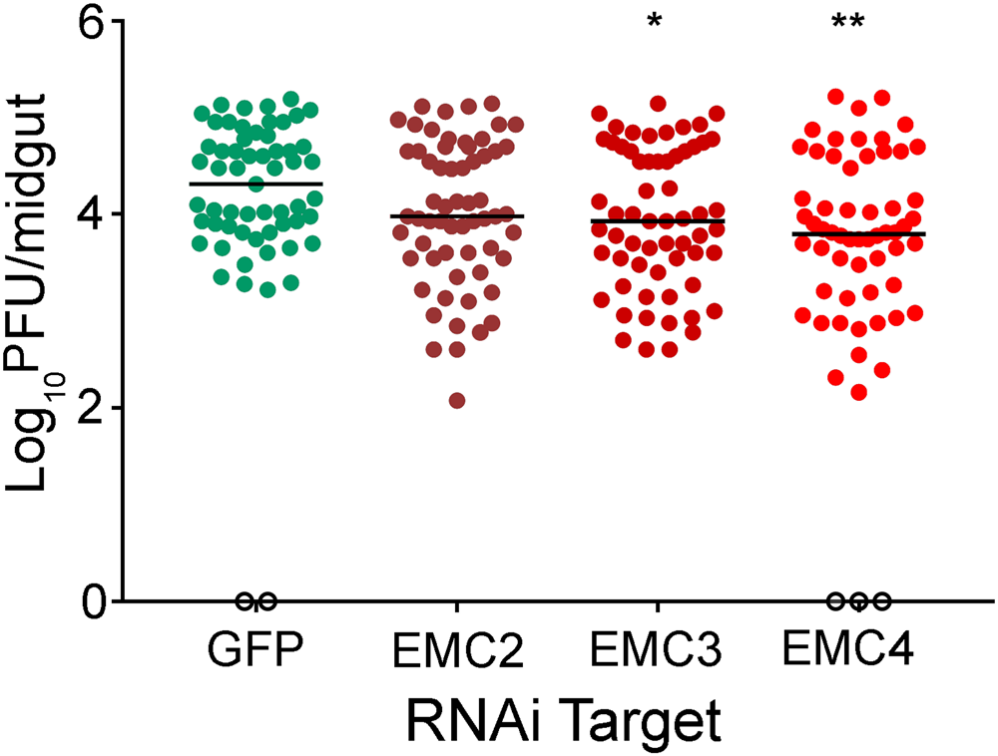
The EMC is required for DENV2 replication in *Aedes aegypti* mosquitoes. Mosquitoes were injected with dsRNA targeting the indicated genes and infected with DENV2 via bloodmeal 3 days later. After seven days of infection mosquitoes were harvested and midguts dissected for virus titration. Each data point represents the titer obtained from a single midgut. P values were calculated using the Mann-Whitney U test (*p < 0.05, **p < 0.001).

*Aedes aegypti* mosquitoes were injected with dsRNAs targeting GFP as a negative control or EMC subunits and infected with DENV2-NGC by ingestion of a virus-containing blood meal (Fig. S2). Fifty-seven (of fifty-nine) mosquitoes injected with dsRNA targeting GFP were productively infected with DENV, with a median of 4.31 log_10_ PFU/midgut (Fig. 4). In the experimental groups, 61 (of 61) mosquitoes treated with dsRNA targeting aaEMC2 and aaEMC3 subunits established productive infections and 57 (of 60) mosquitoes in aaEMC4 subunit. Silencing efficiency was determined at the day of DENV infection. EMC gene expression ranged from 49%, 73%, and 68% for EMC2, EMC3, and EMC4, respectively, compared to GFP-injected controls. The median virus production for mosquitoes treated with dsRNA targeting aaEMC2, aaEMC3 or aaEMC4, was 3.98 log10 PFU/midgut, 3.93 log10 PFU/midgut, and 3.80 log10 PFU/midgut, respectively (Fig. 4). Results show that dsRNA targeting aaEMC3 (P = 0.0257) and aaEMC4 (P = 0.0010) significantly reduced infection, while dsRNA targeting aaEMC2 (P = 0.1115) slightly reduced infection, but was not statistically significant. We also observed high mortality in EMC-silenced group vs. GFP controls suggesting a fitness cost of EMC silencing, reducing the number of mosquitoes with high levels of silencing (data not shown). These results provide the first evidence that the EMC is an evolutionarily conserved DENV proviral host factor for both humans and mosquitoes.

### The EMC is required for efficient virus entry

**Figure 5:**
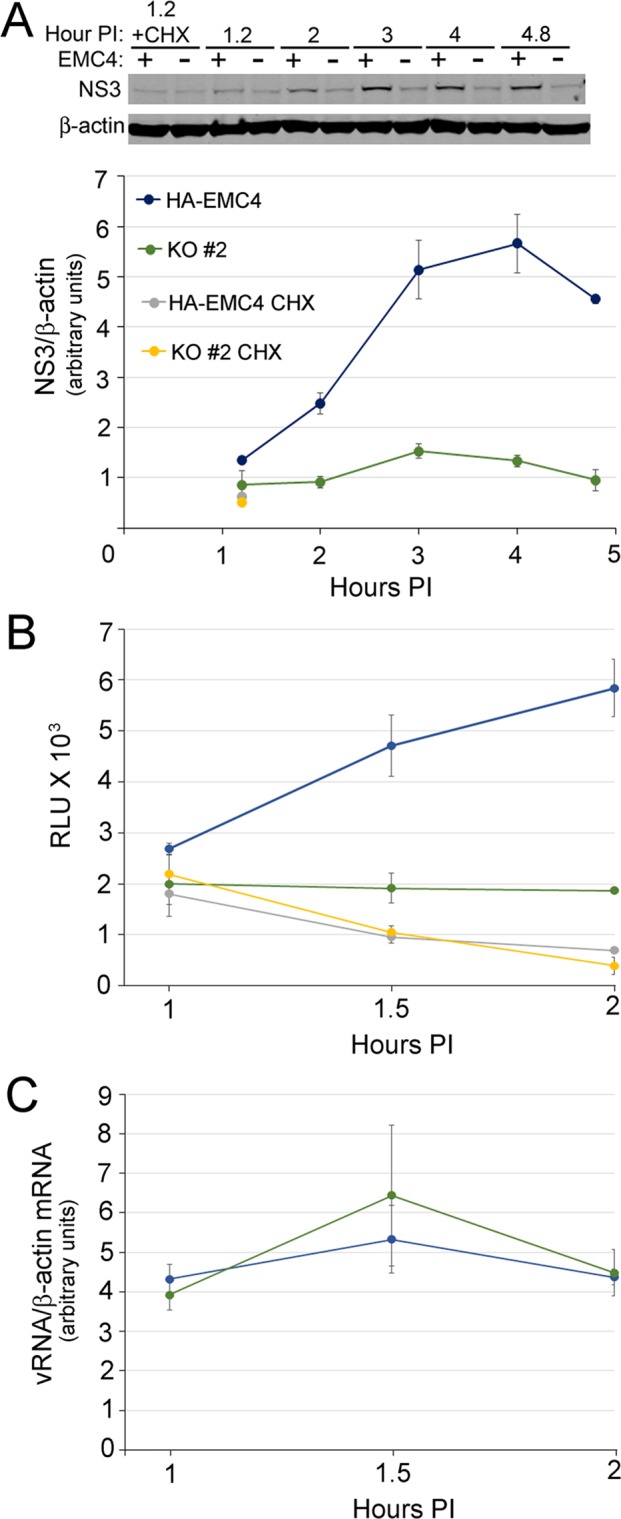
The EMC is required at an early stage of the virus lifecycle. (**A**) The indicated HuH-7 lines were infected with DENV2 at a high MOI (10) and protein lysates were analyzed for viral NS3 levels at the indicated time points after infection. Cycloheximide = CHX. The western blots are from the same gel and the image was cropped to show mature DENV2 NS3 and ß-actin (as loading control). (**B**) The same cell lines as in panel (**A**) were infected with a DENV2 encoding RLuc in the presence or absence of CHX. Cells were infected at an MOI of 5 and protein samples harvested at the indicated time points for luciferase assays. (**C**) Parallel experiments were performed as in panel (B) except RNA samples were collected for analysis of viral genome levels by RT-qPCR.

We set out to investigate the mechanism(s) by which the EMC promotes virus infection. For these experiments we chose to focus on DENV2 and ZIKV as these viruses were both profoundly affected by EMC4 KO (Fig. 3). Brass *et al*. reported that DENV and ZIKV attachment were unaffected by EMC knockdown^19^. In agreement with this, we failed to observe any difference in YFV17D attachment to EMC4 knockout and parental cell lines (Fig. S1). We next evaluated DENV2 NS3 accumulation at early time points after infection. We infected HuH-7(HA-EMC4) and HuH-7(HA-EMC KO clone #2) cell lines with DENV2-NGC (MOI = 10). Cells were pretreated with NITD008, an RNA dependent RNA polymerase (NS5) inhibitor^38^, to inhibit RNA synthesis. To establish the background signal in these experiments we pretreated cells with cycloheximide (CHX) to block synthesis of viral proteins. The NS3/β-actin protein ratio in HA-EMC4 cells increased over the course of 4 hr pi. In contrast, normalized NS3 levels in infected EMC4 KO cells were significantly (p </=0.05) reduced at all time points tested relative to the parental cell line (Fig. 5A). We further analyzed infection of these cells with an infectious DENV2 encoding *Renilla* luciferase (RLuc)^39^. Luciferase levels were monitored at 1, 1.5 and 2 hr pi in the presence or absence of CHX to control for contaminating RLuc present in virus stocks. This analysis revealed significant differences in RLuc signals between the infected HA-EMC4 and EMC4 KO cells (Fig. 5B) without detectable differences in viral RNA levels (Fig. 5C). With CHX, the levels of input RLuc decayed over the time course. Interestingly, in EMC4 KO cells the RLuc signal produced by infection was above the CHX control at 1.5 and 2 hr pi, suggesting that the block imposed by lack of EMC4, although profound, is not absolute. Together, these data indicated that EMC promoted an early stage of DENV2 infection, at or before the step of viral protein biogenesis, as suggested by Brass and colleagues^19^.

We developed a new method to ascertain effects of EMC4 on early steps in the viral lifecycle. This method, termed VIR-CLASP, depends on uncoating of viral genomes and crosslinking of exposed viral genomes to host RNA-binding proteins (RBPs) in the cytoplasm. For this protocol, stocks of ZIKV were prepared in cells labeled with 4-thiouridine (4SU), allowing viral genomes to incorporate 4SU. The thiol group allows for efficient RNA-protein crosslinking upon exposure to UV light at 365 nm. We infected HA-EMC4 and EMC4 KO cells with labeled ZIKV (PRVABC59) at a high MOI (500) for one hour at 4 °C, the cells were washed with cold PBS to remove free virus, further incubated for 30 min at 37 °C, and then irradiated cells with UV_365_ to induce RNA-protein crosslinking. Cells were then lysed and viral ribonucleoprotein complexes stringently isolated (see Materials and Methods) for analysis using solid-phase purification of these complexes under denaturing condition.

**Figure 6:**
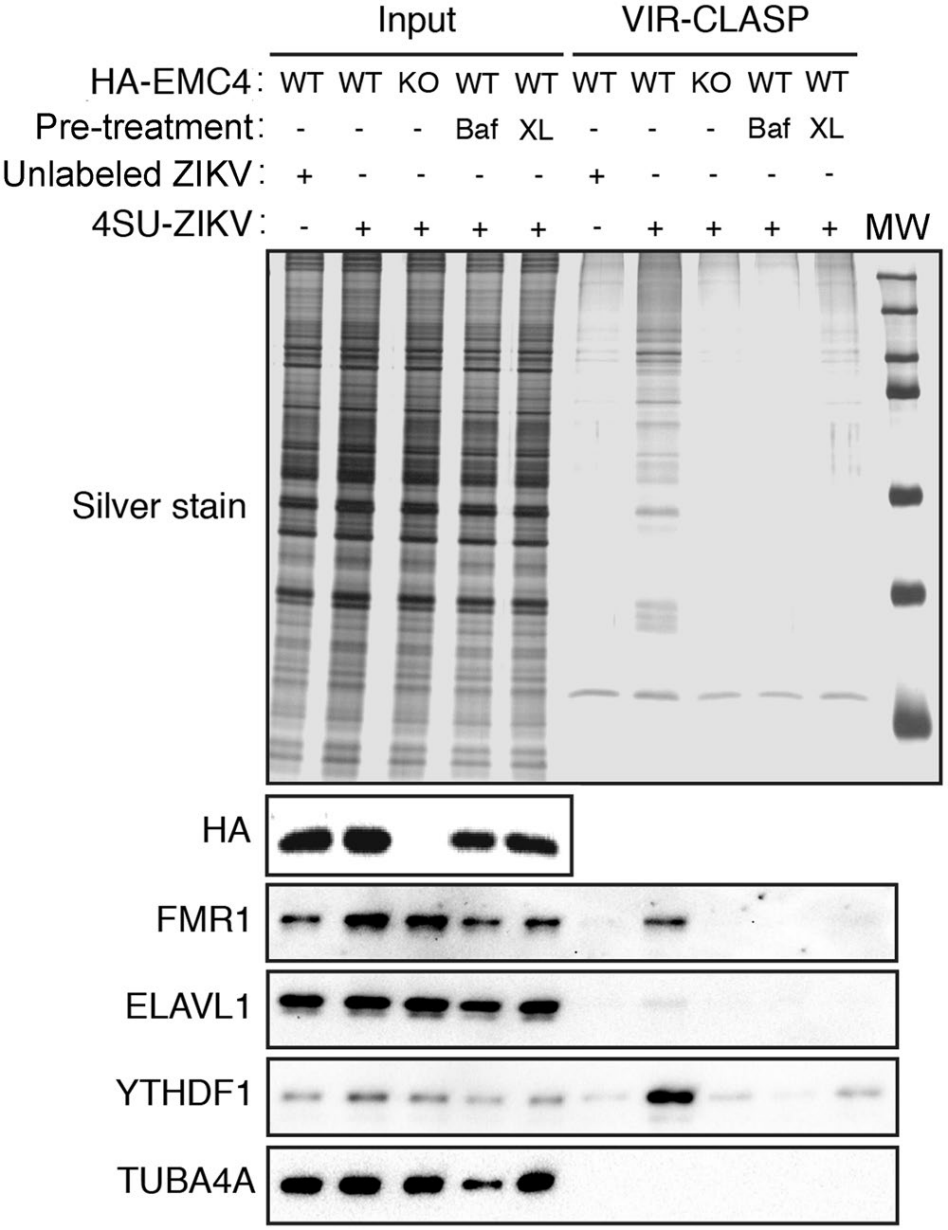
VIR-CLASP demonstrates that EMC4 is required for ZIKV entry and uncoating. SDS-PAGE and silver stain (top), or immunoblot (bottom) of VIR-CLASP performed on HA-EMC4 CRISPR knock-in (WT) or knockout (KO) HuH-7 cells infected with unlabeled or 4SU-labeled ZIKV. Baf, 200 nM bafilomycin for 1 hr; XL, virus was pre-crosslinked with UV_365nm_. The western blots are from the same gel and the image was cropped to show HA-EMC4, FMR1, ELAVL1, YTHFDF1, and TUBA4A.

Three important controls were incorporated into these experiments: (i) infection with unlabeled ZIKV, (ii) pre-treatment of cells with bafilomycin which prevents endosome acidification^40^ and fusion of viral envelope, and (iii) UV irradiation of virus prior to infection. Silver stain analysis revealed multiple proteins crosslinked to ZIKV RNA only in HA-EMC4 cells infected with 4SU-labeled virus, whereas bafilomycin pre-treatment or irradiation of virus prior to infection reduced protein crosslinking as expected (Fig. 6). Importantly, the level of proteins recovered by VIR-CLASP was dramatically reduced in EMC4 KO cells (Fig. 6). We discovered, by Western blot analysis, that the RBPs FMRP, YTHDF1 and to a lesser extent ELAVL1, efficiently came down with ZIKV RNA in HA-EMC4 cells infected labeled virus but not in EMC4 KO cells (Fig. 6). The non-RBP, TUBA4A, was not present in any of the VIR-CLASP samples. These results show that EMC4 is required for ZIKV RNA to access the host cytoplasm and implicate the EMC as a host complex required for efficient virus entry or uncoating.

### After viral entry the EMC is required for efficient ZIKV protein accumulation

**Figure 7:**
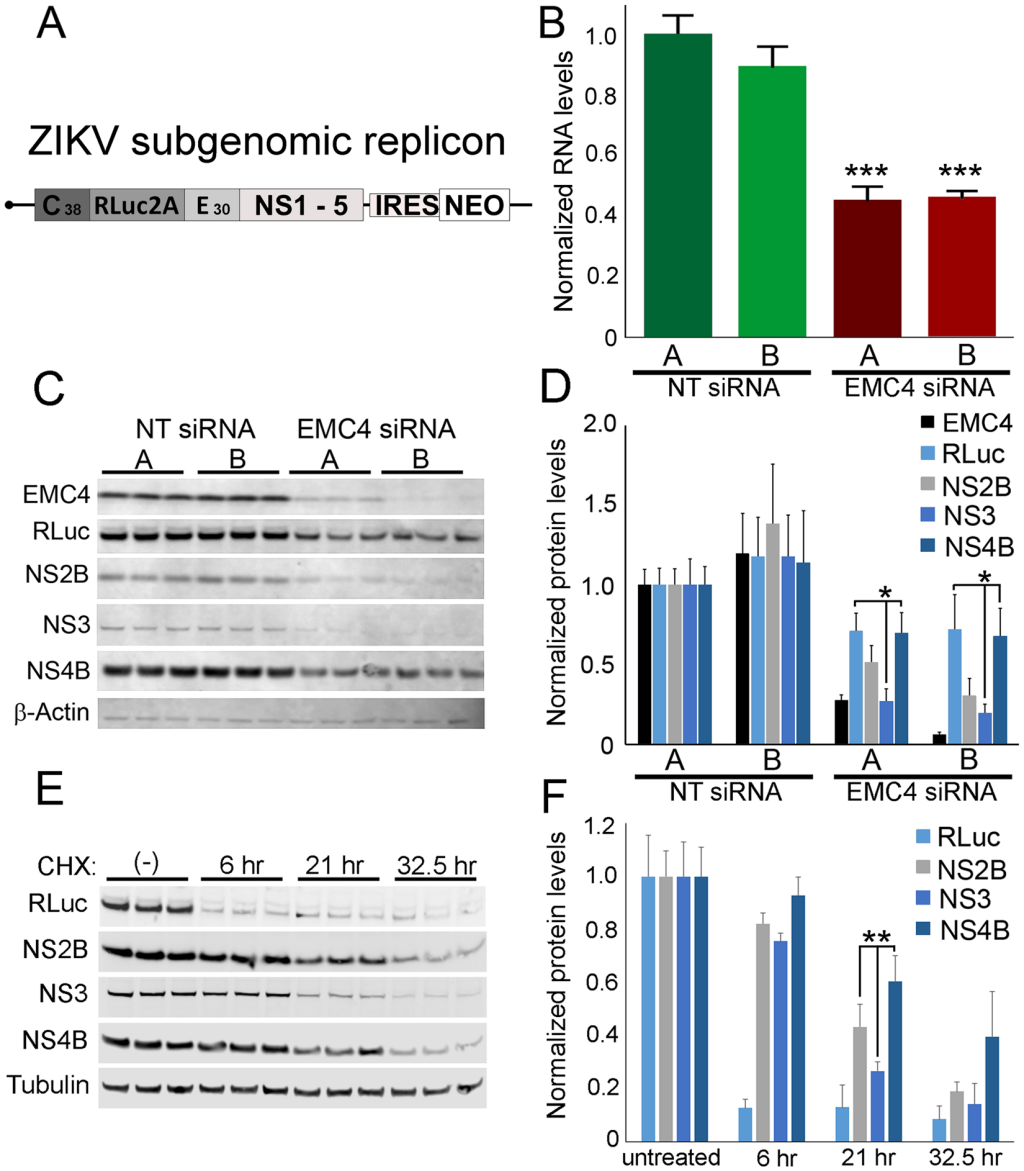
EMC4 is required for ZIKV replicon RNA replication and protein expression. (**A**) Structure of the ZIKV replicon is shown. (**B**) Measurements of replicon RNA levels in cells transfected with non-targeting (NT) control and EMC4 siRNAs. (**C**) Western blot analysis of the indicated proteins is shown from triplicate samples. (**D**) Quantitative analysis of proteins levels from panel (C). (**E**) Cells were treated with CHX for the indicated time points and protein samples harvested. Western blot analysis of the indicated proteins is shown. (**F**) Quantitative analysis of protein levels from two independent experiments performed in triplicate is shown. (*p < 0.05; **p < 0.005). In panels (C,E) western blots are each from one gel and the images were cropped to show mature EMC4, RLuc, and mature viral proteins (NS2B, NS3 and NS4B), and ß-actin or tubulin respectively (as loading controls).

The data presented above, in agreement with work by Brass and colleagues, indicates a likely indirect role for the EMC requirement in a very early viral step, however, given that the EMC has been implicated in protein biogenesis^24,25,27,29^ we wondered whether this complex could also be required for the efficient expression of viral proteins. To test this, we used a HuH-7 cell line harboring an autonomously replicating, subgenomic ZIKV replicon (ZIKV RepNeo)^41^. The replicon includes the ZIKV (strain FSS13025) 5′ UTR, nonstructural proteins and 3′ UTR, which is interrupted by an IRES driven neomycin resistance cassette (Fig. 7A). The structural proteins have been replaced with RLuc, permitting interrogation of viral lifecycle steps related to viral protein biogenesis and RNA synthesis, in the absence of viral entry, assembly and exit.

The ZIKV RepNeo cell line was reverse transfected with either of two independent negative control siRNAs or anti-EMC4 siRNAs. Four days after transfection, the total cell-associated RNA from the RNAi-treated cells was collected and RT-qPCR was used to quantify endogenous GAPDH mRNA and viral replicon RNA. Viral replicon RNA levels for negative control siRNAs A and B were not statistically different, while anti-EMC4 siRNA transfection reduced replicon RNA by 55% and 54% for siRNAs A and B, respectively (Fig. 7B), consistent with an important post-entry role during ZIKV replication.

We also analyzed the expression of several replicon proteins after EMC4 knockdown. The ZIKV RepNeo cell line was reverse transfected siRNAs as above and protein samples were collected four days later for analysis of EMC4, RLuc, NS2B, NS3, NS4B and β-Actin levels. EMC4 protein expression for the negative control siRNAs was similar, while EMC4 protein expression was reduced by 73% or 94% by EMC4 siRNAs A and B, respectively (Fig. 7C,D). Relative to the negative control siRNAs, expression of RLuc and all ZIKV proteins assayed was reduced by EMC4 knockdown, consistent with an important post-entry role for the EMC in ZIKV replication (Fig. 7C,D).

We noted interesting differences in the effects of EMC4 depletion on levels of the analyzed ZIKV proteins and RLuc. Specifically, NS3 levels were reduced more significantly than RLuc or NS4B, while the effect of EMC4 knockdown on NS2B was intermediate (Fig. 7C,D). We reasoned that these differential effects may be related to inherently different rates of turnover for these proteins, which would implicate a role for EMC in the biogenesis of viral proteins. To address this, we analyzed the levels of each protein in CHX-treated cells over the course 32.5 hours (Fig. 7E,F). The ZIKV proteins exhibited different rates of degradation and this was particularly evident at 21 hours post-CHX treatment when 61% of NS4B, 43% of NS2B and 27% of NS3 remained intact (Fig. 7F). The relative stabilities of these proteins correlated with the effects of EMC4 knockdown which had the largest effect on NS3 and smallest effect on NS4B (Fig. 7D). These observations strongly suggest that the EMC promotes the biogenesis of ZIKV proteins. Interestingly, RLuc was the most unstable protein examined as only 13% remained at 6 hours post-CHX treatment (Fig. 7F), but was relatively unaffected by EMC4 depletion (Fig. 7D). The dichotomy between levels of RLuc and levels of the viral proteins after EMC depletion suggests that this complex impacts the biogenesis of viral proteins at a stage subsequent to initiation of translation. Importantly, our data clearly indicate an EMC requirement for a post-entry effect that is fully consistent with the model proposed by Hedge and colleagues^25,26,29^ wherein the EMC coordinates the insertion of transmembrane domains and can protect polytopic proteins from premature degradation.

All of the data presented above leads to the conclusion that EMC is required twice for infection of *Aedes* transmitted flaviviruses, first, at a step subsequent from attachment but prior to viral uncoating, and second, at a step subsequent to uncoating, and very likely a step after translation initiation, but required for the biogenesis of viral proteins.

## Discussion

We screened a library of siRNA pools for the ability to reduce DENV2 infection of a human cell line at a time-point that permitted at least one complete viral lifecycle. We identified DENV proviral factors that were required to a degree similar to the positive control, a subunit of the vATPase. Consistent with the biology of DENV infection^9^, our high confidence hit list included three subunits of the vATPase (ATP6V0C, ATP6V0D1, and ATP6V1F), many subunits of the ribosome and translational apparatus (n = 31), and factors necessary for translocation and processing of the viral polyprotein (SRP54, SEC61A1, SPCS2). Among the high confidence hits there are many that are novel, and mechanistic insights are still not available into how the majority proviral factors, novel and previously known, impact flaviviruses. Nonetheless, this screen, previous ones from our laboratory^15,16^ and those from other groups^17–20,42^ have opened important lines of investigation for these viruses.

A common theme from all the aforementioned screens is a reliance on host factors that reside in or impact the ER. Previously, our lab identified that drosophila fly gene CG33129 and its human homologue TMEM214 as DENV proviral factors^15^, although the mechanistic details were not further pursued. The current screen concurred since TMEM214/FLJ20254 was once again identified as a DENV proviral factor. TMEM214/FLJ20254, although largely unexplored, was reported to localize to the ER and may function in the ER-stress response^43^. The current screen also supports the hypothesis that the TRAPP complex may have a conserved pro-flaviviral role since TRAPPC1 was identified here and in our prior published screen for YFV proviral factors along with TRAPPC11/FLJ12716^16^. TRAPPC4 was also identified in a recent screen for flavivirus proviral factors^19^. Depletion or mutation of TRAPPC11 (and other subunits) disrupted ERGIC and Golgi morphology, prevented export^44^ and N-linked glycosylation of a model protein^45^ suggesting that the mammalian TRAPP complex is involved in ER-to-golgi trafficking and post-translational glycosylation. Further investigation of the DENV-TRAPP complex relationship may inform on the virus-host interaction, the biological role(s) of the TRAPP complex and may broaden our understanding of rare human genetic disorders. ERI3, a recently identified DENV-3′UTR interacting protein, was shown to support viral replication^46^, and appears herein as a DENV proviral factor, demonstrating how orthogonal screening technologies converged on a common DENV proviral factors ER resident signalases mediate specific steps in polyprotein processing^47^, and recent screens identified multiple subunits of the signalase as flavivirus proviral factors^18–20^. The Diamond lab created renewed interest here by suggesting that the composition and activity of the signalase(s) involved in viral polyprotein processing may vary^20^. Our screen identified signalase subunit SPCS2 as a DENV proviral factor, and future investigations may evaluate the SPCS2-dependent signalase activity in the context of viral polyprotein processing. The OST complex is a recently identified pan-flavivius proviral factor^18–20^ that supported viral replication through an enzyme-independent mechanism that remains to be clearly deciphered^18^, and our screen also identified that the OST complex subunits DAD1 and RPN2 are a DENV proviral factor.

Among the most critically required proviral factors we have investigated is the EMC. This ER associated 10 subunit complex is exquisitely required for the replication of DENV2, DENV4, YFV, ZIKV (this work and^16–20^). Like many other proviral factors^15^ we show that EMC is required for viral replication in both human cells and *Aedes aegypti* mosquitoes. The EMC requirement is not shared by WNV. Wu and colleagues reported that WNV infection was only slightly delayed in an EMC2 knockout cell line, relative to the parental cell line^17^. Diamond and colleagues reported that knockout of EMC4 reduced WNV infection 12 hr pi approximately 75% by one sgRNA, but reported a second sgRNA resulted in a statistically insignificant WNV reduction. In addition, WNV was reduced at least two fold for two different sgRNAs targeting EMC6^20^. Although, WNV was not affected by EMC4 knockout in our experiments, it could be that a delay would have been missed in our experiments. Nonetheless, it is clear that there is a marked difference between the EMC requirement for DENV2, DENV4, YFV, and ZIKV versus that for WNV and this difference deserves further investigation.

What are the roles of the EMC on the flaviviral lifecycle? Brass and colleagues reported that DENV and ZIKV attachment were not blocked by EMC knockdown^19^. Nevertheless, the pattern of internalized DENV E protein in DENV infected cells was altered shortly after infection of EMC knockdown cells^19^ which suggested a defect up to and including viral RNA synthesis. For ZIKV, attachment was not altered between control and EMC knockdown cells, however, there was a suggestion that entry was blocked leaving virus on the surface of the cell. Our data using VIR-CLASP, which can directly detect interactions between incoming viral genomes and cellular proteins and thus uncoating, supports a role for the EMC in ZIKV entry. A possible explanation for the effect on entry is that EMC facilitates the biogenesis and/or assembly of required receptors in the plasma membrane or endosome resulting in an indirect effect on viral infection^27,28,48–50^.

An important finding of this study is that the EMC promotes a post-entry phase of the ZIKV lifecycle. Depletion of EMC subunits reduced ZIKV protein biogenesis in a replicon system where virus entry is not required. Our data also suggest that the EMC is acting at a step after translation initiation of the RLuc ORF and self-cleavage of the luciferase from the ZIKV polyprotein. We conclude that the EMC plays a role in the biogenesis of the flaviviral proteins, which requires the synthesis, membrane insertion, modification, and maturation of the polytopic viral polyprotein. This role is consistent with the reported roles of the EMC on cellular proteins^24,25,27–29^. EMC is important for the biosynthesis of multi-pass membrane proteins^26,28^, a function likely mediated by the complex’s transmembrane domain insertase activity^25^. This EMC function appears to be essential to stabilize client polytopic proteins from degradation^29^. Given its structural and topological complexity the flaviviral polyprotein would be expected to directly require EMC. Perhaps the surprise is that WNV manages to circumvent this requirement. Given the difficulty of creating chimeric flavivirus ORFs it will be challenging, but interesting, to use this differential requirement to further delineate the properties that make a nascent protein sensitive to EMC function. The fact that the EMC is required for more than one step in the viral lifecycle can explain the profound inhibition we observe for many flaviviruses.

## Methods

### Genome-scale RNAi screen

The siRNA screen was conducted at the Duke RNAi Screening Facility using the Qiagen Human Genome siRNA Library v1.0. For a detailed description of the methods and materials, see reference^30^. For each host gene, four independent targeting siRNAs were combined into two separate pools named Set AB and Set CD^33^. Lipofectamine RNAiMax (Invitrogen), siRNAs and Optimem (Gibco) were complexed with 1,200 HuH-7 (human hepatoma cell line; gift of Dr. Eckard Wimmer, SUNY Stony Brook) cells/well such that cells were reverse transfected at a final concentration of 15.4 nM siRNA and approximately 0.077% v/v Lipofectamine RNAimax in Dulbecco’s modified Eagle medium (DMEM) supplemented with 5% heat-inactivated fetal bovine serum (FBS), 100 U/mL penicillin-streptomycin (Gibco) (antibiotic) and 0.01 M HEPES. 52 hours post-transfection, DENV2 (New Guinea C strain) (a gift of Dr. Aravinda DeSilva, UNC Chapel Hill) was added to the cells at 1,066 FFU/well (approximately 0.2 MOI). The infection was terminated 42-hours post infection using 4% paraformaldehyde. DENV2 infected cells were fluorescently labeled using the flavi-group specific mouse anti-envelope antibody, 4G2 (produced from ATCC D1-4G2-4-15 (ATCC® HB-112™), and goat anti-mouse AlexaFluor 488-conjugated secondary antibody (Invitrogen). Automated imaging and analysis was performed using the Cellomics Array Scan VTI system. Two fields/well were analyzed and the number of cells and infection rates were calculated.

### Data analysis for genome wide RNAi screen

We selected an analysis strategy in which high-confidence DENV host factors were identified when the biological duplicate siRNA pools each reduced DENV at least as much as a control siRNA that targets a subunit of the vATPase. In order to identify hits, the mean and standard deviation of the % infection for all ATP6V0C siRNAs (Qiagen # SI00307384) from each respective set of the plates (Set AB compared to Set CD) was calculated. The Z-score for each well in Set AB was calculated using the mean and standard deviation from the ATP6V0C control siRNAs for all plates. These Z-scores are presented in Supplemental Table 1. The 455 high confidence hits have Z-scores equal to or less than 3.00.

### Meta-analysis of the DENV and YFV RNAi screen data sets

The genome-wide data sets for the YFV17D^16^ and DENV2 siRNA (this manuscript) screens were collated. Any gene target for which one or more wells from any contributing dataset had low numbers of cells/well (400 and 245 or less over two fields for the YFV and DENV screens, respectively) was filtered from further analysis resulting in removal of 2,034 genes. 20,875 gene targets were analyzed in this meta-analysis. The percent of cells infected by either YFV17D or DENV2 was ranked for each gene. A nonparametric summation of ranks was adopted in order to combine disparate screen datasets. In total, four YFV genome-wide siRNA screen datasets were combined with two DENV datasets. In order to compensate for the four YFV datasets, relative to the two DENV datasets, all rank values for the YFV data sets were divided by 2. Finally, the total screen ranks were summed for each of the 20,875 genes with a minimum possible sum rank of 4 and maximum of 83,500. A mock permutation of the ranks followed by summation of the permuted ranks (n > 1 × 10^7^ permutations and summations) was performed and identified that a p ≤ 0.00137 was equal to a summation of 13,100. The calculated false discovery rate for this p-value is < 30 genes.

### Cell lines

African green monkey VERO cell line (American Type Culture Collection #CCL-81) and the human hepatoma HuH-7 cell lines were maintained at 37 °C, 5% CO_2_ in DMEM supplemented with 10% FBS and antibiotic. HuH-7 (ZIKV RepNeo) cell line (kindly provided by Pei-Yong Shi, UTMB) was maintained in 0.3 mg/mL Geneticin (Gibco). C6/36 (American Type Culture Collection #CRL-1660) cell line was maintained at 28 °C, 5% CO_2_ in RPMI supplemented with 10% heat inactivated fetal bovine serum and antibiotic.

### Viruses

DENV2 (strain New Guinea C), DENV4 (TVP360) (gift of Dr. Aravinda DeSilva, UNC, Chapel Hill, USA), and YFV Asibi (TVP10101; kindly provided by Dr. Robert Tesh; UTMB Arbovirus Reference Collection) were propagated on C6/36 cells. YFV17D (a gift of Dr. Charles Rice, Rockefeller University) and ZIKV (strain P6-740) (TVP12663; kindly provided by Nikos Vasilakis, UTMB) were propagated on Vero cells. Cleared, virus-containing cell culture media were used to inoculate cell cultures for viral infection experiments. WNV (strain NY99 (35262-11) was obtained from BEI Resources and provided by the Duke University Regional Biocontainment Laboratory. DENV2-RLuc virus was a kind gift of Dr. Pei-Yong Shi (UTMB)^39^.

### Biosafety statement

All experiments using pathogenic YFV Asibi and WNV were performed per approved protocols at the Duke University Regional Biocontainment Laboratory in a Biosafety Level 3 environment. All other experiments involving infectious virus were conducted at Biosafety Level 2 at Duke University or the University of Texas Medical Branch.

### Plasmids

Plasmids used in the gene editing experiments were described in and obtained from Addgene: pcDNA3.3-TOPO CAS9, pcDNA3.3-TOPO CAS9_D10A, pCR Blunt II TOPO U6 (sgRNA cloning vector), pCR Blunt II TOPO U6 GFP T1 and pCR Blunt II TOPO U6 GFP T2.

The sgRNAs targeting EMC1, 4 and 5 were designed using sequences predicted and published in reference^34^. EMC1 was targeted by sequence human_exon_crispr_v1_003453. EMC4 was targeted by sequences human_exon_crispr_v1_120084 and human_exon_crispr_v1_120085. EMC5 was targeted by sequences human_exon_crispr_v1_188532 and human_exon_crispr_v1_188533. The sgRNAs targeting EMC3 target the sequences 5′-CCGCCACTACGTGTCCATCCTGC-3′, 5′-CCACTACGTGTCCATCCTGCTGC-3′, and 5′-CCTGCTGCAGAGCGACAAGAAGC-3′. The plasmids expressing sgRNAs that target EMC1, 3, 4, and 5 were constructed following the procedure outlined as “Option B” in the “gRNA Synthesis Protocol” in^34^.

### Gene editing

Briefly, HuH-7 cells grown on 12-well plates were transfected with 0.5 μg of pcDNA3.3-TOPO CAS9 and 0.5 μg of the sgRNA cloning vector using Lipofectamine 2000 (LF2K) (Invitrogen) following manufacturer’s recommendations. The following day, transfected cells were passaged into 10 cm dishes containing growth media plus 1 mg/mL G418. Cells were carried in G418-containing media for 3–5 days after which cells were cultured in media without G418 for an additional 7 days. The pools of edited cells were seeded onto collagen-coated 96-well plates (Corning COSTAR) at a density of 2 × 10^4^ cells/well prior to infection.

To generate HuH-7 (HA-EMC4) cell lines, parental HuH-7 cells were transfected as above with 0.08 pmol pcDNA3.3-TOPO-CAS9_D10A, 0.17 pmol sgRNA # human_exon_crispr_v1_120084 plasmid and 0.10 pmol dsDNA donor (IDT). The dsDNA donor included flanking arms ~350 bp upstream and downstream of the HA-insertion.

After transfection cells were cultured as described above and then passaged onto collagen coated 15 cm plates at sparse density. After 12 to 14 days colonies were selected by eye and transferred to individual wells in 96 well format. HA-tagged colonies were initially screened by replica plating the 96 well plates and staining one plate using primary anti-HA antibody (Cell Signaling). Subsequent SURVEYOR (Transgenomic) assays and amplicon sequencing confirmed knockin of the HA-tag was inserted into EMC4 loci.

HuH-7(HA-EMC4 KO) cell lines were derived from a common parental HuH-7(HA-EMC4) cell line by co-transfection with pcDNA3.3-TOPO CAS9 and sgRNA # human_exon_crispr_v1_120085. Candidate knockout colonies were identified using an analogous procedure as described above with the exception that HA-negative colonies were selected for expansion. Subsequent amplicon sequencing and enzymatic SURVEYOR assays confirmed that the HA-tag contained frame shift mutations.

### Virus infection in 96 well format

The cell monolayer was infected with YFV17D at an MOI 0.1 in 30 μL DMEM, 5% FBS, 0.01 M HEPES and antibiotic for 1 hr at 37 °C rocking the plate every 15 min. After 1 hr, virus containing media was removed, monolayers were rinsed 1x with phosphate buffered saline with magnesium and calcium (PBS with Mg/Ca). The infected monolayers were incubated 38 hours post infection in fresh media (DMEM, 5% FBS, 0.01 M HEPES and antibiotic) until the termination of the experiment.

### Virus production assays

Cell lines were seeded onto 48 well assay plates in growth media. The following day, cells were inoculated at the indicated MOI at 37 °C for approximately 1 hour. After virus absorption, cells were rinsed 1x with PBS plus MgCa, 0.25 ml DMEM (5% FBS, antibiotic, 0.01 M HEPES) was added. At the indicated time post absorption, virus containing media was collected and stored at −80 °C. Virus production was quantified by focus formation assay as previously described^15^.

YFV-Asibi and WNV NY99 were similarly titered except that infected cells were labelled with the mouse anti-YFV envelope protein antibody (EMD/Millipore, clone 2D12.A, 1:2000) or mouse anti-WNV envelope protein antibody 7H2 (VRL-Maryland; 1:2000). HRP-conjugated secondary antibodies were diluted 1:2000 in blocking buffer and incubated over night at 4 °C. Finally, the foci were developed using TrueBlue peroxidase substrate (KPL) following manufacturer’s recommendations.

### RNAi-mediated EMC subunits silencing in *Aedes aegypti* mosquitoes

RNAi-mediated gene silencing, mosquito infection and plaque assays were carried out as previously described^37,51,52^. Briefly, *Aedes aegypti* mosquitoes Rockefeller strain (Johns Hopkins University) were maintained on 10% sucrose solution at 27 °C and 80% relative humidity with a 14:10 h light:dark cycle. Three- to four-day old female mosquitoes were cold-anesthetized and injected with 200 ng of dsRNA per mosquito targeting EMC2, EMC3, or EMC4 and mosquitoes injected with GFP dsRNA were used as controls. The dsRNA was synthesized using the HiScribe™ T7 *In Vitro* Transcription Kit (New England Biolabs). The primer sequences (5′-3′) used for dsRNA synthesis are the following (lowercase letters correspond to the T7 polymerase promoter site): EMC2 Forward-taatacgactcactatagggGGAATATCTTCCGAAAGTGGC, Reverse-taatacgactcactatagggCGCATTAGTTTCGTCCTTTTT, EMC3 Forward-taatacgactcacttagggCTGGGTGTTTCTGCCCATAG, Reverse-taatacgactcactatagggACGTTGATGAAGTTACCCTTGA, EMC4 Forward-taatacgactcactatagggCTGTCACTTGCAATCGTGTG, Reverse-taatacgactcactatagggTTTCAGGATCAGGTGGCTTT. At 3 days post-dsRNA injection, mosquitoes were infected with a blood meal containing DENV2 NGC at 10^6^ PFU/ml via a membrane feeder. Midguts were dissected and individually collected at 7 days post-blood meal, homogenized, and the supernatants were used to perform a plaque assay and determine plaque-forming units (PFU) per midgut. Data presented are a pool of four independent biological replicates, and P-values were determined with the Mann-Whitney U test.

Gene silencing was determined at 3 days post-injection of EMC’s dsRNA or GFP as control group. RNA extracted from five whole mosquitoes per biological replicate were analyzed by qRT-PCR. The ribosomal protein S7 gene was used to standardize and verify gene silencing using the following primers: EMC2-F 5′-TTCGGGAACTGTGCGACTAC-3′, EMC2-R 5′-GAAGGCCGCTTTTGCGTATT-3. ECM3 F 5′-TTCTTGAACGTGTTCGGCCT-3′, ECM3-R 5′-CTCCCGACATCTGATCCTGC-3′. EMC4 F 5′-GGATTGGTTGGCCTTTGCAG-3′, EMC4-R 5′-CTCGCGAAAGAACCCCCTAA-3′. S7-F 5′-GGGACAAATCGGCCAGGCTATC-3′, S7-R 5′-TCGTGGACGCTTCTGCTTGTTG-3′.

### Immunofluorescent detection of viral antigens

Cells were fixed using 4% paraformaldehyde followed by permeabilization using 0.1% Triton-x-100 (Sigma) in PBS. Cells were rinsed 3X with PBS and 0.1% Tween-20 (wash buffer) and blocked for at least 1 hour at room temperature with 1% Normal Goat Serum diluted in wash buffer (blocking buffer). Primary antibody (mouse anti-dsRNA (J2, English Scientific), 1:4000; mouse anti-flavivirus E protein (4G2), 1:2000; mouse anti-yellow fever envelope protein (clone 2D12.A), 1:2000) was diluted in blocking buffer and applied for either 1 hour at room temperature or overnight incubation at 4 °C. Following incubation, the cells were rinsed 3X with wash buffer for at least 15 minutes per wash at room temperature. Secondary antibody was diluted in blocking buffer and incubated with the cells for at least 1 hour at room temperature. In addition, Hoechst stain (Sigma) was added during incubation with the secondary antibody. Fluorescent secondary antibodies (Goat anti-mouse AlexaFluor 488, goat anti-mouse AlexaFluor 647 (Invitrogen), were used at 1:2000 dilution. Following incubation, the cells were rinsed three times with wash buffer and stored in PBS.

### RNAi knockdown of EMC4

The HuH-7 (ZIKV RepNeo) cell line was reverse transfected with siRNAs targeting EMC4 or negative controls. Lipofectamine RNAiMax, siRNA duplex and Opti-mem were complexed with cells following manufacturers’ recommendations at final concentrations of 0.15% Lipofectamine RNAimax and 10 nM siRNA in growth media. NT siRNA A and B correspond to Qiagen AllStars Negative Control siRNA (cat# 1027231) and Dharmacon ON-TARGETplus Control siRNA (cat# D-001810-01-20), respectively. EMC4 siRNA A and B correspond to Dharmacon ON-TARGETplus human TMEM85 siRNA cat# J-021126-05 and Dharmacon ON-TARGETplus human TMEM85 siRNA cat# J-021126-06, respectively. The cells were incubated for approximately 96 hours before total RNA or total protein was collected for subsequent analysis.

### RT-qPCR and virus binding assay

The virus-binding assay was performed as previously described^53^ except that cells were infected at MOI of 10 and incubations were carried out for one hour. Total cell-associated RNA was isolated by Trizol (Life Technologies) extraction following manufacturer’s recommendations. Reverse transcription was performed on 250 ng RNA using the Applied Biosystems High Capacity cDNA RT Kit (Thermo-Fisher). Quantitative PCR was performed using cDNA equivalent to 1/100 of the starting RNA material. Endogenous GAPDH (Forward primer: 5′-AGCCACATCGCTCAGACAC-3′; Reverse Primer: 5′-GCCCAATACGACCAAATCC-3′), YFV17D (Forward primer: 5′-ATTTGGGCGAAGGAGTATCCCAGT-3′; Reverse Primer: 5′-ACGCTAACCAGCATCATCAGGAGT-3′) or ZIKV (Forward primer: 5′-CTGTGGCATGAACCCAATAG-3′; Reverse Primer: 5′-ATCCCATAGAGCACCACTCC-3′) cDNAs were amplified the Applied Biosystems Power SYBR Green PCR Master Mix (Thermo-Fisher) and a StepOnePlus real-time PCR system.

### Western blotting

Cells were solubilized in 2% n-Dodecyl beta-D-maltoside (Sigma), 1% NP40 (IGEPAL; Sigma), 200 mM KCl, 20 mM HEPES, with protease inhibitor cocktail (Roche). Samples were heated to 70 °C for 10 minutes in NuPAGE LDS (Thermo-Fischer) sample buffer with 2.5% 2-mercaptoethanol (Acros). Gel electrophoresis was performed on total protein cell lysate under denaturing conditions using the Invitrogen NuPAGE 4–12% Bis-Tris Gel with either NuPAGE MOPS SDS (Life Technologies) or MES SDS (Life Technologies) running buffers. Proteins were transferred (NOVEX NuPAGE transfer buffer) overnight onto either 0.45 micron nitrocellulose (BioRad) or 0.2 micron PVDF (BioRad) membranes and blots were blocked using Starting Block (Thermo Scientific). Blots were incubated with appropriate primary antibodies Renilla Luciferase (Abcam), 1:2500; Zika NS2B (Genetex), 1:2500; Zika NS3 (Genetex), 1:2000; Zika NS4B (Genetex), 1:4000; EMC2 (SantaCruz), 1:500–1:750; EMC4 (Abcam), 1:4000; β-Actin (SantaCruz), 1:4000; and α-tubulin (Thermo-Fischer), 1:2000 diluted in Starting Block overnight at 4 °C then washed 3 over 1 hour at room temperature with PBS with 0.1% tween20. The next day, appropriate secondary antibodies (Donkey anti-rabbit 800 (LICOR), Donkey anti-mouse 680 (LICOR), Donkey anti-mouse 800 (LICOR) were incubated at 1:20000 dilution in Starting Block and washed as before. The antibody signals were imaged and quantified using the Li-Cor Odyssey CLx imaging system.

### VIR-CLASP

Zika virus (ZIKV; strain: PRVABC59; cat# VR-1843) and Vero cell line (African green monkey (Cercopithecus aethiops) kidney normal cell line; cat# CCL-81) were obtained from ATCC. ZIKV was propagated in Vero cells with or without 5 mM 4-thiouridine (4SU). Virus stocks were purified by ultracentrifugation of clarified supernatants through a 20% sucrose cushion in TNE buffer (50 mM Tris-HCl [pH 7.2], 0.1 M NaCl, and 1 mM EDTA) at 125,000 × *g* for 4 hr in a Beckman SW32Ti rotor. To remove remaining free 4SU, virus pellets were washed three times with TNE buffer, and then resuspended in virus dilution buffer (DEME medium containing 10 mM HEPES [Gibco] supplemented to contain 1% FBS), aliquoted, and stored at −70 °C. Virus titers were determined by plaque assay using Vero cells.

For VIR-CLASP, cells were infected with 4SU labeled virus for 1 hr at 4 °C and uninfected virus was washed away with cold PBS. The infected cells were incubated for 30 min at 37 °C, prior to 365 nm ultraviolet irradiation. Irradiation using UV_365nm_ allows for covalent crosslinking of interacting host proteins to the incoming RNA genome but not to cellular RNAs not labeled with 4SU. To irradiate with UV_365nm_, the growth medium was removed and washed with PBS. Cells were irradiated on ice with 365 nm UV light (0.6 J/cm2 × 2 times) in a Stratalinker 2400 (Stratagene). Cells were scraped off in 2.5 ml PBS per plate.

Cells were lysed in denaturation buffer (50 mM Tris–HCl, pH 6.8, 10% glycerol, 2.5% SDS, 0.66% NP-40), incubated for 10 min at 95 °C and subsequently slowly cooling them to 25 °C. Crosslinked RNA-protein complexes were purified by Solid-Phase Reversible Immobilization (SPRI)^54^ beads (GE Healthcare, cat# 65152105050250) under denaturing SPRI buffer (50 mM Tris–HCl, pH 6.8, 10% glycerol, 2.5% SDS, 0.66% NP-40, 1 M NaCl, 8% PEG-8000). This allows for quantitative recovery of RNA and enrichment of crosslinked proteins since only those proteins can be pulled down under high SDS conditions. To each sample, 0.66× (e.g. 660 μl of beads for 1 ml of sample) of SPRI beads were added. The SPRI beads and complexes were washed 5 times with denaturing SPRI buffer. The crosslinked RNA-protein complexes were eluted in denaturation buffer. To reduce non-specific binding on the beads, SPRI purification was repeated. An equal volume of 4x Benzonase buffer (80 mM Tris-HCl, pH 7.5, 600 mM NaCl, 20 mM MgCl_2_, 4 mM DTT, 40% Glycerol) and 2x volume of water were added to eluted samples, followed by the addition of Benzonase (EMD Millipore, cat# 70746-4) to a final concentration of 50 U/ml, and incubation for 2 hr at 37 °C. Proteins were precipitated by methanol and chloroform and then re-suspended in 2x NuPAGE LDS Sample Buffer (Thermo-Fisher, cat# NP0007) with 50 mM DTT.

Antibody to YTHDF1 (anti-YTHDF1; 17479-1-AP) was from Proteintech; anti-ELAVL1 (ab200342) and anti-TUBA4A (ab7291) were from abcam; Antibody to FMR1 (MAB2160) was from Millipore; anti-HA.11 Epitope Tag (901501) was from biolegend. Samples were separated by SDS-PAGE. After electrophoresis, proteins were semi-dry transferred (Bio-Rad) to nitrocellulose membranes (Hybond-ECL, GE Life Science). Protein membranes were taken through a standard immunoblot protocol followed by enhanced chemiluminescent detection (Luminata Forte ECL, Millipore) using a chemiluminescence imaging system (ChemiDoc MP, Bio-Rad).

### Availability of materials

Materials, data and associated protocols described in this manuscript will be promptly available to readers.

## Supplementary information

supplementary information

Table S1

Table S2

## Data Availability

All data generated or analyzed during this study are included in this published article (and its Supplementary Information files).
