# Supplementary material for: Dual roles for the ER membrane protein complex in flavivirus infection: viral entry and protein biogenesis
Source: Sci Rep. 2019 Jul 4;9:9711. doi: 10.1038/s41598-019-45910-9 (PMC6609633; doi:10.1038/s41598-019-45910-9)

SUPPLEMENTARY INFORMATION

Dual roles for the ER membrane protein complex in flavivirus infection: viral entry and protein biogenesis

Nicholas J. Barrows[1,2], Yesseinia Anglero-Rodriguez[3,¶], Byungil Kim[4,¶], Sharon F. Jamison[1], Caroline Le Sommer[1], Charles E. McGee[5], James L. Pearson[1], George Dimopoulos[3], Manuel Ascano[4], Shelton S. Bradrick[1,2,*], and Mariano A. Garcia-Blanco[1,2,6,*]

[1]Department of Microbiology and Molecular Genetics, and Center for RNA Biology, Duke University, USA

[2]Department of Biochemistry and Molecular Biology, University of Texas Medical Branch, USA

[3]W. Harry Feinstone Department of Molecular Microbiology and Immunology, Johns Hopkins Bloomberg School of Public Health, USA

[4]Department of Biochemistry, Vanderbilt University, USA

[5]Duke Human Vaccine Institute, Duke University, USA

[6]Programme of Emerging Infectious Diseases, Duke-NUS Medical School, Singapore

[¶]These authors contributed equally to this work.

*Correspondence to Shelton S. Bradrick (ssbradri@utmb.edu) or Mariano A. Garcia-Blanco (maragarc@utmb.edu)

Supporting Information Legends

Figure S1. Image of full length gels for critical data. The only cropped gel where it is essential to show a full length gel is the one showing that EMC4 KO cell lines do not have a band that cross reacts with EMC4. One representative western blot of a full length gel is shown. DU12C3 is HA-EMC4 #2, DU13A1 and DU13C4 are HA-EMC4-KO #1 and #2 respectively. EMC1, EMC4, HA-EMC4, ß-actin, and a non-specific band (*) are indicated.

Figure S2. Silencing efficiency of EMC2, EMC3 and EMC4 in *Ae. aegypti*. EMC2, EMC3 and EMC4 RNA remaining three days after dsRNA injection using qRT-PCR. Bars represent SEM of three biological replicates relative to RNA levesl in mosquitoes injected with GFP dsRNA, which were used as a negative control for all experiments.

Figure S3. EMC4 knockout does not affect YFV-17D attachment. The indicated cell lines were incubated with YFV-17D at a M.O.I. of 10 on ice for 1 hr to allow attachment, then washed with PBS and total cell associated RNA was extracted. Alternatively, cell lines were incubated with YFV-17D at a M.O.I. of 10 on ice for 1 hr to allow attachment, then washed, and fresh media was added followed by a 1hr 37°C incubation and finally the cells were iced, treated for 3 min with high-salt alkaline solution total RNA was collected. YFV-17D genomes were measured by quantitative RT-PCR and normalized to GAPDH mRNA. Mean and standard deviation are presented.

Figure S4. Image of full length gels for other data. All uncropped gel images except the critical one shown in Figure S1 above. Panels A-D indicate the main figure that is connected to the uncropped images.

Table S1. Loss of function screen for DENV2-NGC host factors. All of the numerical data for the loss of function screen is presented in this table. Sheet 1 shows data for all of the control siRNAs. Sheet 2 shows data for the two sets (AB and CD) of two siRNAs in the screen both in terms of cell numbers (or valid object counts) and in terms of % infection (or % selected). Sheet 3 shows shows data that was used for analysis in the manuscript. See Methods and Results for a description of filters used to obtain these data. Sheet 4 shows the hits as described in the Results.

Table S2. Comparison of host factors for yellow fever virus (17D) and DENV2-NGC. A permutation analysis of the ranks of the YFV and the DENV2 screens identified common 94 antiviral and 274 proviral factors. Large ribosomal subunit proteins in red font and small subunit proteins in purple font.

Date: WB 12/10/2014 (25ug tot prot/Ln)	X	X	X	X	X
M.W. Ladder	X				
HuH-7 (WT)		X			
DU12C3			X		
DU13A1				X	
DU13C4					X

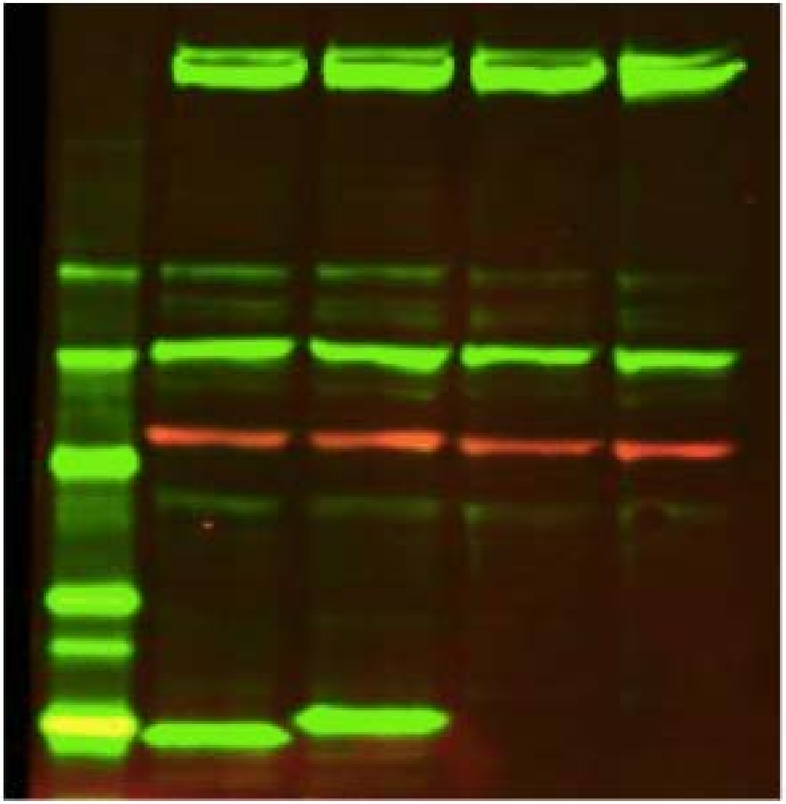

Figure S1

Figure S2

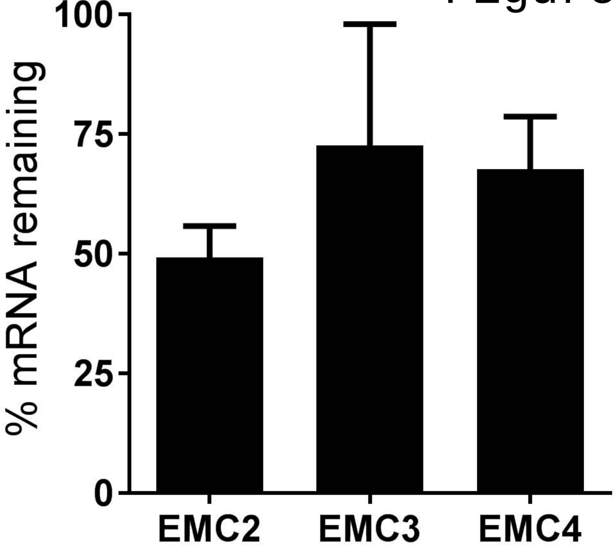

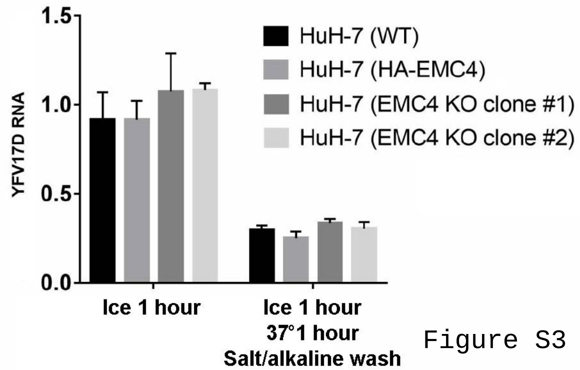
1.5
1.0
0.5
0.0
YFV17D RNA
HuH-7 (WT)
HuH-7 (HA-EMC4)
HuH-7 (EMC4 KO clone #1)
HuH-7 (EMC4 KO clone #2)
Ice 1 hour
Ice 1 hour
37°1 hour
Salt/alkaline wash

Figure S3

Figure S4A

For Figure 5A

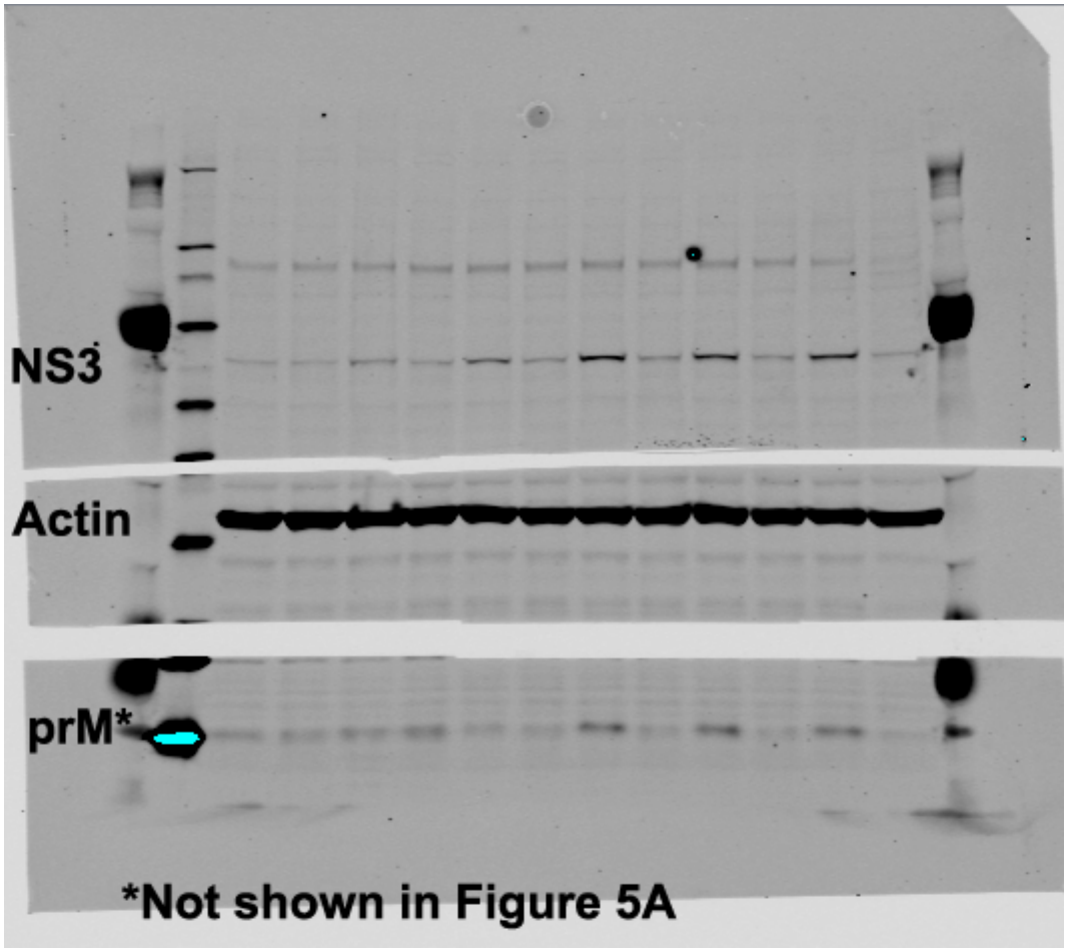

Figure S4B

For Figure 6

Raw data of Figure 6

Silver stain

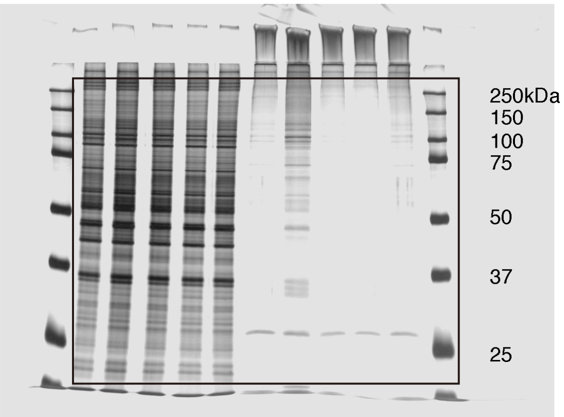

IB HA-EMC4

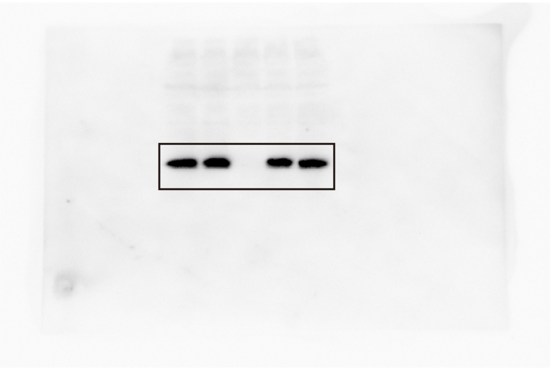

IB FMR1

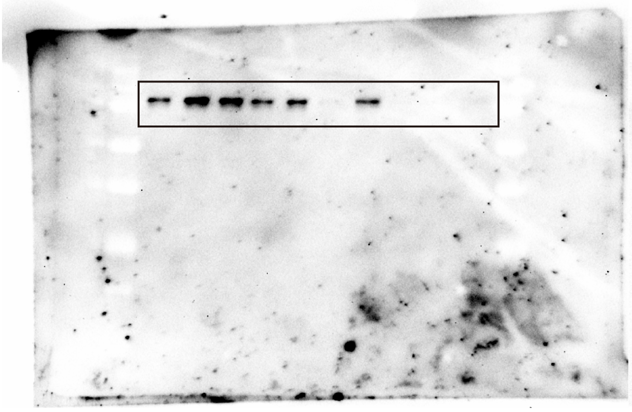

ELAVL1

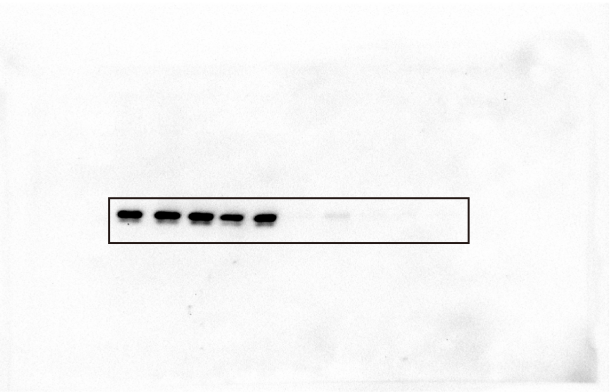

YTHDF1

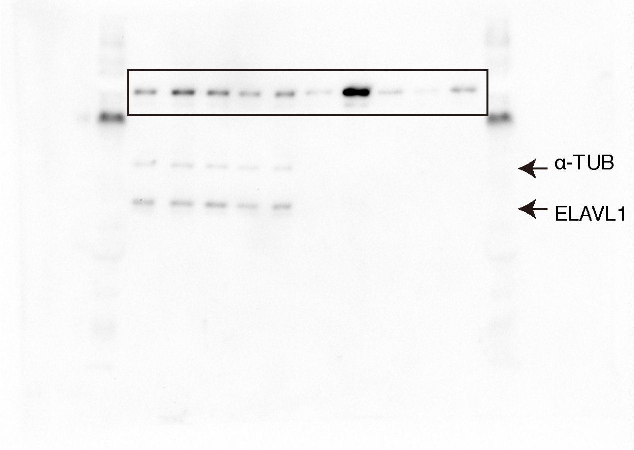

α-TUB

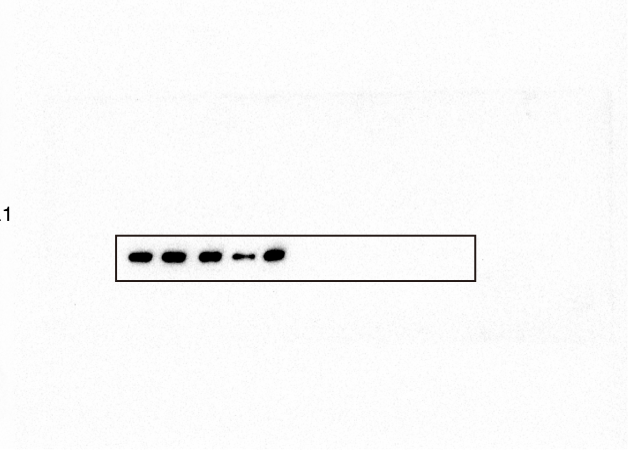

Figure S4C

For Figure 7C.

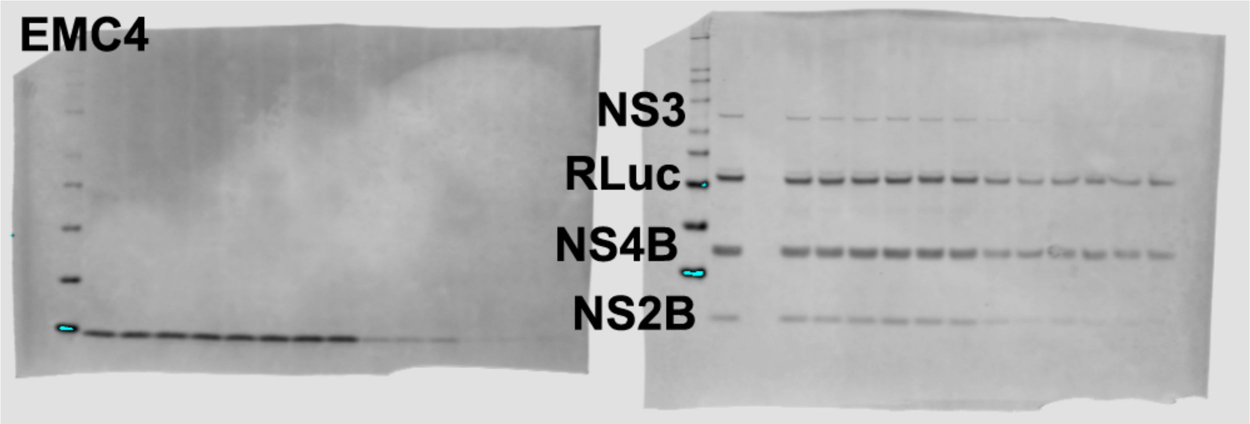

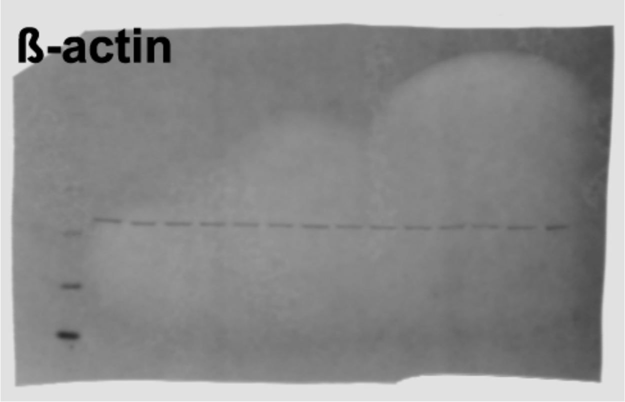

Figure S4D

For Figure 7E

RLUC

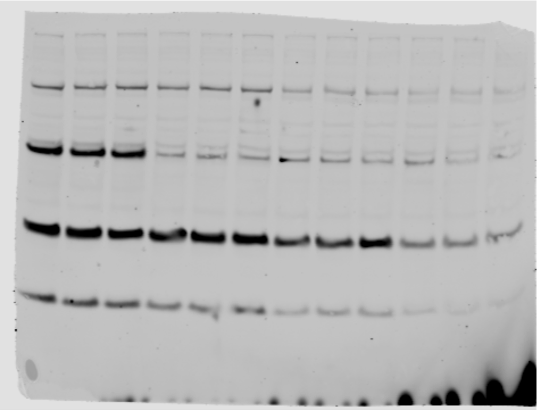

NS3

NS2B

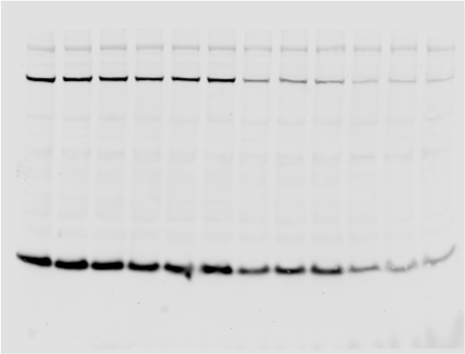

NS4B

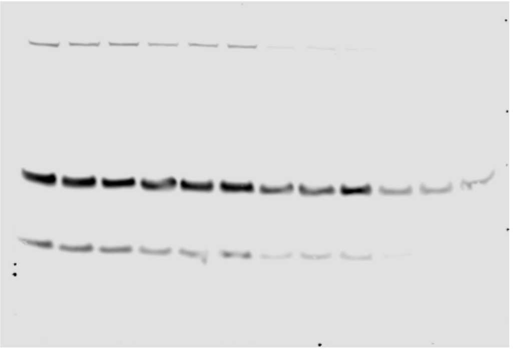

tubulin

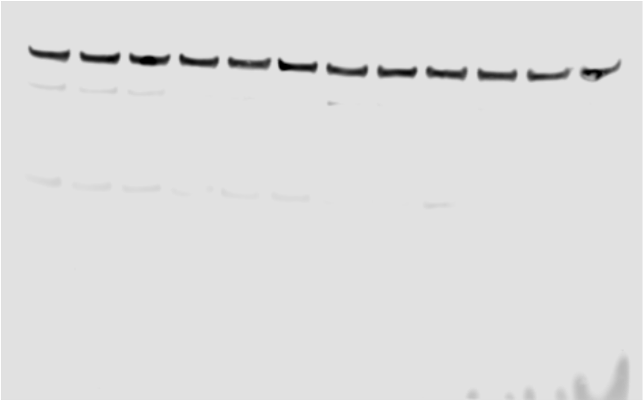

Supplement: Supplementary file 1 — supplementary information [file 41598_2019_45910_MOESM1_ESM.pdf]
